# 

**Published:** 1900-10-13

**Authors:** 


### NOTICE TO CORRESPONDENTS.

All MSS., Letters, Books for Review, and other matters intended for the Editor should be addressed The Editor, "The Hospital," The Hospital Building, 28 & 29 Southampton Street, Strand, W.C.

The Editor cannot undertake to return rejected MS., even when accompanied by stamped directed envelope.

### Notice to Advertisers and Subscribers.

All Advertisements, Orders for Copies of The Hospital, and Business Communications should be addressed to **THE MANAGER (Not to the Editor),** The Hospital, The Hospital Building, 28 & 29 Southampton Street, Strand, London, W.C.

The Cost of Subscription, post free, is as follows:—Per week, 2½d.; per per quarter, 2s. 8d.; per half-year, 5s. 3d.; per annum, 10s. 6d. Foreign.—Per annum, 15s. 2d., payable, in all cases, in advance.

**NOTICE.—Vol. XXVIII. of "The Hospital" (April to September, 1900), in handsome cloth binding, will shortly be on sale at the Office of this Journal, price, 7s. 6d. Cases for binding the Numbers contained in this Volume 1s. 6d. Index to Vol. XXVIII., 2½d., post free.**

**The Monthly Part for October is now ready. Price 6d., by post 8d.**

### The Student of Medicine.

### APPOINTMENTS.

Anna L. Church, M.D., R.U.I., appointed Medical Superintendent to the Lady Aitchison Hospital, Lahore, Punjab. H. S. Cooper, M.R.C.S., L.R.C.P.Lond., appointed Certifying Factory Surgeon for the Yaxley District of the County of Huntingdon. W. Crawtree, B.Sc.Vict., M.B., Ch.B., appointed Medical Officer for the Workhouse of the Stoke-upon-Trent Union. Richard Lane Joynt, F.R.C.S., appointed Surgeon to the Meath Hospital, Dublin. James Marsh, M.B., C.M.Edin., appointed Certifying Factory Surgeon for the Urban District of Tyldesley and Atherton in the County of Lancaster. Ernest W. Martin, M.B., Ch.B., appointed Resident Medical Officer to the City of London Hospital for Diseases of the Chest, Victoria Park. J. O'Donoghue, L.R.C.P., L.R.C.S.I., appointed Medical Officer for the Howth Dispensary District. W. R. Pollard, M.R.C.P.Edin., L.R.C.S.I., appointed Medical Officer for the Blackburn Union Cottage Homes. Robert Riddell, M.B., C.M.Edin., appointed Certifying Factory Surgeon for the Sandbach District of Cheshire. Robert H. Ross, L.R.C.P., L.R.C.S.Edin., appointed Medical Officer to the Whitehead Constabulary. W. R. Smith, M.D.Lond., appointed Medical Officer for the Beeston and Wollaton District of the Basford Union. H. T. Wright, L.R.C.P., L.R.C.S.Edin., appointed Medical Officer of Health for the No. 2 District of Alston. Owen L. Rhys, M.B., Ch.B.Edin., appointed Assistant House Physician to the Cardiff Infirmary.

### Scraps and Gleanings.

The Protene Company announce that they have opened a branch at 141 Regent Street, W.

The sum collected this year on Hospital Saturday for the Essex and Colchester Hospital amounted to £115 odd.

During last year the number of in-patients treated at the Fleetwood Cottage Hospital was 70, as against 60 for the previous year and 53 for the year 1897-98.

The total amount collected this year up to September 28th by the Woking Hospital Saturday collection was £237 odd, which exceeds last year's collection by about £15, and again creates a record in Surrey.

The Nantwich Rural Council have unanimously adopted a scheme providing for the erection of a joint isolation-hospital to contain 16 beds, 12 for the rural and four for the urban district.

The Aylesbury and District Friendly Society had a very satisfactory report to present at their final meeting held recently. From this report it appears that £90 odd has been received this year, compared with £56 last year and £76 in 1894—the largest sum collected till this year.

At the recent annual meeting of the Fleetwood Cottage Hospital a resolution was passed to furnish a room in the hospital for the use of patients as a convalescent ward, as a memento of the beginning of the twentieth century. This resolution is conditional upon funds being raised for the purpose, outside the ordinary sources of revenue.

The Working Men's committee in connection with the Hull Royal Infirmary passed a resolution at a recent meeting to the effect that they could best serve the cause of the infirmary by binding themselves together and inaugurating some effort for raising a substantial amount for the Building Fund, and to increase their ordinary donations.

At the recent quarterly meeting of the Gloucester Infirmary Governors the Chairman explained the grounds on which increased support was asked from the subscribers and general public. The infirmary was not in debt (although reports had been circulated that it was), and there were sufficient funds forthcoming from its annual subscriptions and donations, with the interest on its endowments, to do the work for which it was originally intended, i.e. to maintain about 87 beds. But of late years they had been obliged to increase their accommodation to about 100 beds, and it was to provide for the maintenance of this increased accommodation that more funds were required.

At a recent meeting of the Hull Corporation Finance Committee it was decided to agree to the application contained in a letter from Sir James Reckitt, viz. that the subscription from the Corporation to the Hull Royal Infirmary should be increased from 25 guineas to £100. It was pointed out at this meeting, however, that the present annual subscription of the Corporation was in reality considerably more than 25 guineas, in view of the fact that the Hull Infirmary was only rated at the nominal value of £40 instead of its real ratable value of £564, the rates upon which would amount to £175. The water also was supplied to the infirmary at less than the ordinary scale by about £40 a year.

